# Dual Effects Exerted *in Vitro* by Micromolar Concentrations of Deoxynivalenol on Undifferentiated Caco-2 Cells

**DOI:** 10.3390/toxins7020593

**Published:** 2015-02-16

**Authors:** Gina Manda, Mihaela Andreea Mocanu, Daniela Eliza Marin, Ionelia Taranu

**Affiliations:** 1 Cellular and Molecular Medicine Department, “Victor Babes” National Institute of Pathology, 99-101 Splaiul Independentei, Bucharest 050096, Romania; E-Mail: miha_moc@yahoo.com; 2 Laboratory of Animal Biology, National Institute for Research and Development for Biology and Animal Nutrition, Calea Bucuresti No. 1, Balotesti, Ilfov 077015, Romania; E-Mails: daniela.marin@ibna.ro (D.E.M.); ionelia.taranu@ibna.ro (I.T.)

**Keywords:** deoxynivalenol, Caco-2 cells, cellular impedance, cell proliferation, cell adhesion

## Abstract

Contamination of crops used for food and feed production with *Fusarium* mycotoxins, such as deoxynivalenol (DON), raise important health and economic issues all along the food chain. Acute exposure to high DON concentrations can alter the intestinal barrier, while chronic exposure to lower doses may exert more subtle effects on signal transduction pathways, leading to disturbances in cellular homeostasis. Using real-time cellular impedance measurements, we studied the effects exerted *in vitro* by low concentrations of DON (0.37–1.50 μM), relevant for mycotoxin-contaminated food, on the proliferation of undifferentiated Caco-2 cells presenting a tumorigenic phenotype. A 1.5 μM concentration of DON maintained cell adherence of non-proliferating Caco-2 cells, whilst arresting the growth of actively proliferating cells compared with control Caco-2 cells *in vitro*. At 0.37 μM, DON enhanced Caco-2 cell metabolism, thereby triggering a moderate increase in cell proliferation. The results of the current study suggested that low concentrations of DON commonly detected in food may either limit or sustain the proliferation of colon cancer cells, depending on their proliferation status and on DON concentration. Soluble factors released by *Lactobacillus strains* can partially counteract the inhibitory action of DON on actively proliferating colon cancer cells. The study also emphasized that real-time cellular impedance measurements were a valuable tool for investigating the dynamics of cellular responses to xenobiotics.

## 1. Introduction

Deoxynivalenol (DON), a trichothecene mycotoxin produced by members of genus *Fusarium*, is an important toxic contaminant of crops used for food and feed production (wheat, grains, barley, *etc.*), thus impacting farm animals and humans [1,2,3]. A European large-scale survey conducted on 40,000 food samples evidenced their contamination with DON at important levels (91–5000 μg/kg), varying depending on the country [4]. DON is heat-stable during food processing and hence persistent throughout the food chain [5]. Chronic dietary exposure of humans to DON was estimated to be on average between 0.22 and 0.58 μg/kg of body weight (b.w.) per day, whilst chronic exposure of farm animals was estimated between 3.9 and 43.3 μg/kg b.w. per day [2].

Exposure to high concentrations of DON may alter the intestinal barrier function and morphology, induce vomiting, decreased food intake, and anorexia [6,7,8]. Lower concentrations of DON can impact the intestinal and systemic immune response either directly or indirectly by increased bacterial passage through the intestinal epithelial layer. DON-triggered immune dysfunctions have major implications for human health in terms of inflammation and susceptibility to infection [3,9,10,11]. Based on the existing toxicology and exposure data, the Joint FAO/WHO Expert Committee on Food Additives (JECFA) proposed for DON a provisional maximum tolerable daily intake (PMTDI) of 1 μg/kg b.w. per day [12].

Research efforts have focused on investigating the gastrointestinal effects of DON and their systemic consequences. Most *in vitro* studies examined the impact of DON on intestinal barrier integrity, DON absorption by differentiated intestinal epithelial cell lines, protein synthesis and signal transduction pathways [13,14,15]. However, these studies were conducted on normal epithelial cells when exposed to cytotoxic concentrations of DON (>30 μM) rather than to non-cytotoxic concentrations (<2.5 μM) that are commonly found in food [2,16].

The aim of our study was to investigate in real-time the effects exerted *in vitro* by low concentrations of DON (0.37–1.5 μM) on the proliferation of the human epithelial colorectal adenocarcinoma cell line, Caco-2, in various experimental conditions (non-proliferating *versus* actively proliferating cells). We also investigated if a culture supernatant of a mixture of *Lactobacillus* strains (LB) could modulate the response of DON-treated Caco-2 cells. The study was mainly based on real-time electric impedance measurements using the xCELLigence system. Whenever necessary, alternative methods were used, like tetrazolium salt reduction by metabolically active cells. The current study showed that DON may affect colon cancer cells at dosages achievable in human food products, and that soluble factors released by *Lactobacillus* strains can interfere with this action.

## 2. Results and Discussion

We studied the effects exerted by 0.37–1.50 μM DON on undifferentiated tumorigenic Caco-2 cells that were or were not actively proliferating [17]. These low DON concentrations are relevant for the intake of mycotoxin-contaminated food [15], and do not alter the intestinal barrier permeability, as shown in human Caco-2 and intestinal porcine epithelial cells *in vitro* [18]. Impedance changes associated to cellular adhesion or spreading, as well as cell number were investigated in real time using the xCELLigence technology [19]. Tetrazolium salt reduction was used for determining cellular viability and metabolism.

### 2.1. The Effect of DON on Non-Proliferating Caco-2 Cells

**Figure 1:**
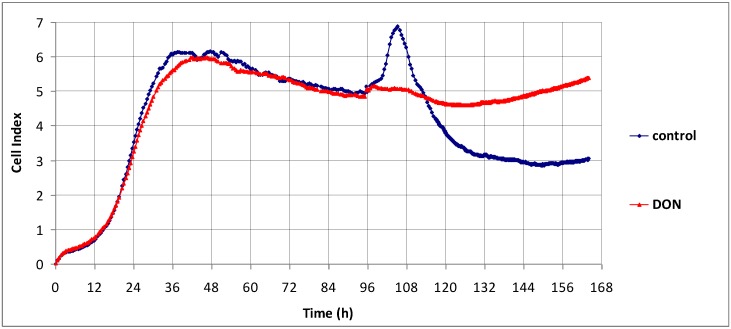
The effects exerted *in vitro* by 1.5 μM deoxynivalenol (DON) on the impedance of confluent Caco-2 cells, measured using the xCELLigence platform. Cells were seeded in E-plates and were allowed 96 h to reach confluence. Culture medium was then changed and DON was added. Values represent the mean cell index of duplicate samples run simultaneously.

Real-time electric impedance measurement was used to investigate the effect of DON on the adherence of non-proliferating confluent Caco-2 cells (Figure 1). Caco-2 cells reached a non-proliferating state in approx. 40 h. Cellular impedance slowly decreased thereafter, as the confluent layer tended to detach from the solid plate surface (macroscopic observation). Replacement of cell culture medium at 96 h triggered a short pulse of increased impedance, as cells ran out of nutritional elements during the previous long-term culture. Impedance decreased progressively thereafter, as the Caco-2 layer continued to detach slowly.

DON was added at 96 h and reinforced the adherence of the non-proliferating Caco-2 layer, especially at longer incubation time, as shown by constant or increased cellular impedance (Figure 1). DON possibly altered the junctions between cells [12,18] and therefore adhesion of loosely connected individual cells to solid support was partly restored. We cannot rule out that DON might also deliver proliferation signals to confluent colon cells [20]. This action of DON might be beneficial for intestinal wound repair [21], but not for tumor progression.

### 2.2. The Effect of DON on Actively Proliferating Caco-2 Cells

**Figure 2:**
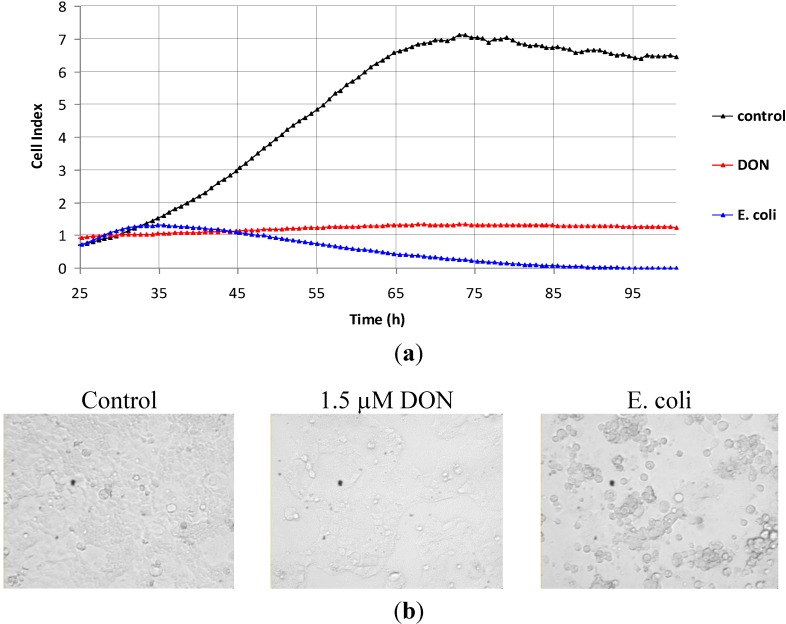
The effects exerted *in vitro* by 1.5 μM DON on the impedance of actively proliferating Caco-2 cells, measured using the xCELLigence platform. Opsonized killed *E. coli* (1 × 10^7^ bacteria*/*mL) were used as cytotoxic agents. Cells were seeded in E-plates and were allowed to adhere for 24 h. Culture medium was then changed and DON or *E. coli* was added. DMSO diluted 1/333 was considered as control. (**a**) Cellular impedance data—representative experiment from seven independent experiments; (**b**) images of Caco-2 cells after 72 h-treatment with DON or *E. coli* (200×).

DON had a different pattern of action on actively proliferating Caco-2 cells. Cells were cultivated for 24 h to allow adhesion, and thereafter 1.5 μM DON was added. Within 6 h after addition to Caco-2 culture, DON started to reduce cellular impedance in comparison with the untreated control (Figure 2). In the presence of DON, cellular impedance was maintained constantly low for a prolonged time (around 120 h), almost at the value registered before DON was added. DON reduced to (41% ± 14%) the impedance values of proliferating Caco-2 cells. While maximal cell index value of non-treated Caco-2 cells was 5.35 ± 0.89, the corresponding cell index value of DON-treated Caco-2 cells was 2.13 ± 0.61 (*p* < 0.01, *n* = 7 independent experiments).

Whilst cellular impedance remained constantly low under DON exposure, impedance decreased progressively towards zero when proliferating Caco-2 cells were treated with a cytotoxic agent (opsonized *Escherichia coli)*. We previously observed by optical microscopy using trypan blue that 1 × 10^7^ opsonized bacteria*/*mL triggered the death and complete detachment of Caco-2 cells in 60–72 h. This cytotoxic effect of *E. coli* was faithfully reflected in the impedance curve depicted in Figure 2. As such, the unchanged impedance values of proliferating Caco-2 cells indicated that treatment with 1.5 μM DON almost totally restricted cell multiplication either by onset of dynamic proliferation/death equilibrium, or by arresting cell cycle [20].

The MTS reduction test was used to measure by an alternative method the decrease of cell number induced by DON. The test is assimilated to a cell proliferation assay, as NADPH or NADH produced by dehydrogenase enzymes in metabolically active cells bioreduce the tetrazolium compound MTS [22]. In 72 h, there was a significant (46% ± 5%) reduction in the number of metabolically active Caco-2 cells (*n* = 4 independent experiments; *p* = 0.021), suggesting that the change in cellular impedance associated with DON exposure was related to cell metabolism or viability.

**Figure 3:**
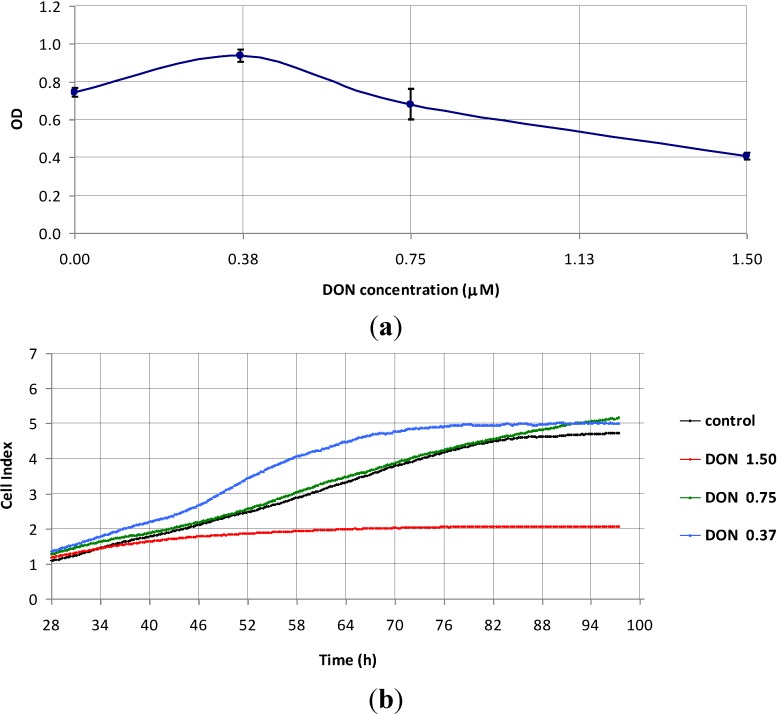
Dose-dependent effects exerted *in vitro* by DON on actively proliferating Caco-2 cells. (**a**) Data obtained by the MTS reduction test (mean values ± SD for triplicate samples ran simultaneously); OD = optical density; (**b**) Representative data obtained using cellular impedance measurements. Tests were performed in parallel. Culture medium was considered as control.

Both the MTS reduction test (Figure 3a) and cellular impedance measurements (Figure 3b) indicated that concentrations of DON below 1.50 μM did not affect Caco-2 cell proliferation (0.75 μM), and there was a trend to increase cellular impedance and metabolism at 0.37 μM. Consequently, DON may exert distinct effects on the proliferation of Caco-2 cells, depending on its concentration (Figure 3) or proliferation potential (Figure 1 and Figure 2). The tight concentration range, in which DON triggered pro- and anti-proliferative signals to tumorigenic Caco-2 cells, highlights a potential risk of colon cancer due to chronic exposure to DON-contaminated food [23].

### 2.3. The Action of Lactobacillus Supernatant on DON-Treated Caco-2 Cells

As LB proved to be promising probiotics to alleviate the deleterious action of *Fusarium* mycotoxins [24], we studied the effect of 1.5 μM DON in the presence of soluble factors released by a mixture of LB (LB sup).

**Figure 4:**
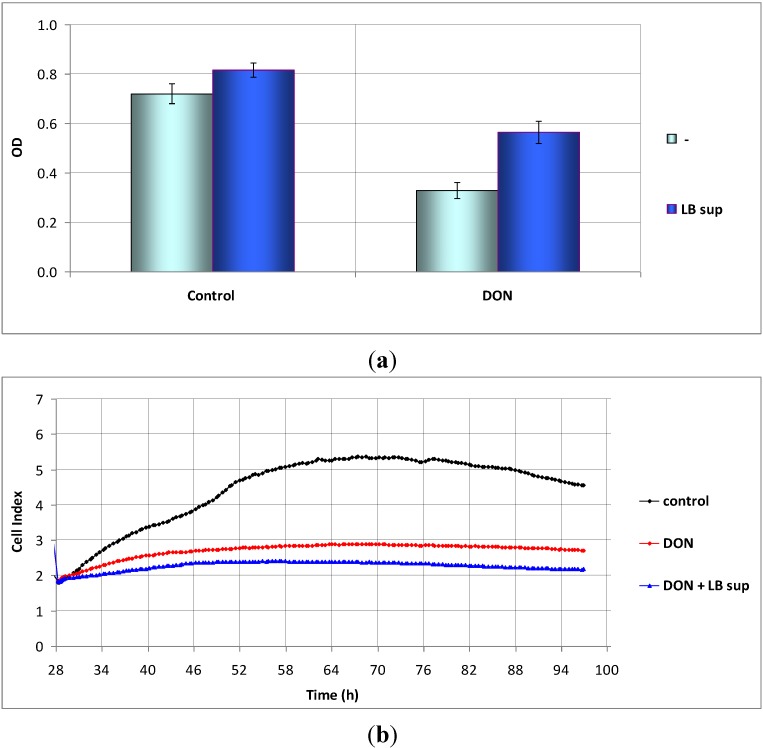
The action of a *Lactobacillus* supernatant (LB sup, 3.5% *v*/*v*) on Caco-2 cells exposed *in vitro* to 1.5 μM DON. (**a**) Data obtained by the MTS reduction test (mean values ± SD, *n* = 4 independent experiments); (**b**) Data obtained using cellular impedance measurements—representative experiment from three independent experiments. Dimethylsulfoxide (DMSO) diluted 1/333 was considered as control. OD = optical density.

LB sup (3.5% *v*/*v*) enhanced to 159% MTS reduction by Caco-2 cells exposed to 1.5 μM DON (Figure 4a, *p* = 0.021), whilst having no significant effect on cellular impedance (example in Figure 4b). Results suggest that LB sup enhanced the metabolic activity of DON-treated Caco-2 cells, responsible for the tetrazolium salt reduction, but did not elicit enhanced cell adhesion or proliferation. Accordingly, we found that the MTS reduction test and cellular impedance measurements might provide complementary information on cell metabolism and adhesion/cell multiplication.

## 3. Experimental Section

### 3.1. Mycotoxin

DON was diluted in dimethylsulfoxide (DMSO, Sigma-Aldrich, St. Louis, MO, USA) at a concentration of 500 μM, aliquoted and kept frozen (−18 °C) until use. Working solutions were prepared in DMEM/F12 culture medium (Life Technologies, Gibco, Grand Island, NY, USA), just before addition to cell cultures. The highest final dilution of DMSO in cultures was 1/333.

### 3.2. Lactobacillus Supernatant (LB Sup)

*Lactobacillus* strains (*Lactobacillus plantarum* (ID1253), *Lactobacillus paracasei* (ID13239), *Lactobacillus acidophilus* (ID11692)) and the DeMan Rogosa and Sharpe medium (MRS) were kindly provided by Dr. O. Dracea from “Cantacuzino” National Research Institute of Microbiology and Immunology, Bucharest, Romania. They were grown anaerobically in MRS for 16 h at 37 °C, then diluted 1:15 in fresh MRS and cultivated another 4 h until mid-log phase [25]. After optical density (OD) measurement (600 nm = 1.0 ± 0.1, corresponding to 1.0 × 10^9^ CFU/mL-*L. plantarum*; 2.9 × 10^8^ CFU/mL-*L. paracasei* and 3.2 × 10^8^ CFU/mL-*L. acidofilus*), bacteria were harvested by centrifugation at 4000 rpm for 10 min at 4 °C. Supernatants were collected and equal volumes were mixed (LB sup). LB sup was filter-sterilized (0.22 μm), aliquoted and kept frozen until use (−80 °C). Just before experiments, aliquots of LB sup were defrozen and centrifuged at 4000 rpm for 10 min at 4 °C.

### 3.3. E. coli

Stabilized and opsonized *E. coli* stock suspension provided in the BurstTest kit (Glycotope Biotechnology, Heidelberg, Germany), containing approx. 1 × 10^9^ bacteria/mL.

### 3.4. Cells

The human colon carcinoma cell line Caco-2 was used in all experiments. The cell line, obtained from ATCC (ATCC^®^ HTB-37™, Manassas, VA, USA), was provided by Dr. Ionelia Taranu, INCDBNA, Balotesti, Romania. Caco-2 cells were grown in DMEM/F12 culture medium supplemented with 20% fetal bovine serum (FBS, Life Technologies, Carlsbad, CA, USA) and antibiotic-antimycotic solution (Life Technologies, Gibco, Grand Island, NY, USA), as recommended by ATCC. The supplemented culture medium will be further named “complete culture medium”. Cells were split twice a week, by detachment with 0.25%/0.02%Trypsin/EDTA (Life Technologies, Gibco, Grand Island, NY, USA) and re-seeding in 25 cm^2^ flasks (40.000 cells/cm^2^). Cells from passages 4 and 26 were used in experiments. Cell viability was assessed by the trypan blue exclusion test (0.4% trypan blue solution, Sigma-Aldrich, St. Louis, MO, USA). Only cell suspensions with viability >95% were used in experiments.

### 3.5. Cell Cultures

Caco-2 cells were used for experiments 48 h after the last passage. 7000 cells were seeded either in 96 well plates for the MTS reduction test or in E-plates (ACEA BIOSCIENCES Inc., San Diego, CA, USA) for cellular impedance measurements. The final sample volume was 100 μL, and 150 μL respectively. For investigations on actively proliferating cells, Caco-2 cells were allowed 24 h to adhere and DON was thereafter added. Confluent non-proliferating Caco-2 cells were obtained in 96 h-cultures before DON addition. Control samples contained DMSO diluted 1/333 in complete culture medium, corresponding to DMSO content in cultures treated with 1.5 μM DON. Duplicate cultures were made in the same day for real-time cellular impedance measurements and triplicate cultures were made for MTS reduction. Independent experiments were performed using cells from different passages (passage number ranging from 4 to 26).

### 3.6. Real-Time Cellular Impedance Measurements

Real-time cellular impedance measurements were performed using the 3 × 16 xCELLigence Real Time Cell Analysis platform (ACEA BIOSCIENCES Inc., San Diego, CA, USA). The electrical impedance across biocompatible microelectrodes placed at the bottom of plate wells was measured. Briefly, E-plates were equilibrated in complete culture medium and impedance was measured for assessing the background signal. Cell cultures were performed as described above, in duplicate. Electric impedance was continuously recorded every 30 min before DON addition and every 15 min thereafter. Data were processed automatically by the xCELLigence dedicated software (version 2.0.0.1301, ACEA BIOSCIENCES Inc., San Diego, CA, USA, 2010) and were displayed as cell index. The cell index is a dimensionless parameter reflecting the relative change in measured electrical impedance reflecting cell status; when cells are not present or not adhered on the electrodes, the cell index is zero. The mean cell index value of duplicate samples was calculated for smoothing cell growth differences in duplicate wells, and was plotted against time.

### 3.7. The MTS Reduction Test

The MTS reduction test was used for assessing the number of metabolically active cells (CellTiter 96^®^ AQ_ueous_ One Solution Cell Proliferation Assay kit, Promega Corporation, Madison, WI, USA). The detection reagent is a tetrazolium compound [3-(4,5-dimethylthiazol-2-yl)-5-(3-carboxymethoxyphenyl)-2-(4-sulfophenyl)-2H-tetrazolium, inner salt; MTS] and an electron coupling reagent (phenazine methosulfate). Cell cultures were performed as described above, in triplicate. Samples containing only complete cell culture medium, supplemented where appropriate with LB sup, were used for background reference. At the end of cell cultivation, 20 μL MTS reagent were added and samples were further incubated for 3 h. OD of samples (OD_S_) and corresponding background (OD_B_) were measured using an ELISA reader (Sunrise from Tecan , Salzburg, Austria), at 490 nm against the 620 nm reference wavelength. Results were expressed as OD (OD = OD_S_ − OD_B_).

### 3.8. Statistic Analysis

Data from the MTS reduction test were presented as mean values ± standard deviation (SD). Comparison between data from independent experiments was performed by the Mann-Whitney test using the SPSS software (version 19, IBM Corp. Armonk, NY, USA, 2010). *p* < 0.05 designates statistical difference between groups.

## 4. Conclusions

We showed that low concentrations of DON, relevant for mycotoxin-contaminated food intake, might exert dual effects on the multiplication of undifferentiated tumorigenic Caco-2 cells, depending on DON concentration and the proliferation status of cells. The results of the current study suggested that DON may slow the progression of actively proliferating colon tumors and that soluble factors released by *Lactobacillus* strains can alleviate the inhibitory action of DON.

We emphasize that real-time cellular impedance measurements is a useful tool for detailing cellular responses to stressors. This approach generally provides similar, though more detailed information than the tetrazolium salts reduction tests. In certain cases, these methods may offer complementary insights into cellular metabolism, proliferation and adhesion.

## References

[1] Rotter B.A., Prelusky D.B., Pestka J.J. (1996). Toxicology of deoxynivalenol (vomitoxin). J. Toxicol. Environ. Health.

[2] European Food Safety Authority (2013). Deoxynivalenol in food and feed: Occurrence and exposure. EFSA J..

[3] Ghareeb K., Awad W.A., Böhm J., Zebeli Q. (2014). Impacts of the feed contaminant deoxynivalenol on the intestine of monogastric animals: poultry and swine. J. Appl. Toxicol..

[4] Schothorst R.C., van Egmond H.P. (2004). Report from SCOOP task 3.2.10 “Collection of occurrence data of Fusarium toxins in food and assessment of dietary intake by the population of EU member states”—Subtask: Trichothecenes. Toxicol. Lett..

[5] Pestka J.J., Smolinski A.T. (2005). Deoxynivalenol: Toxicology and potential effects on humans. J. Toxicol. Environ. Health B.

[6] Goyarts T., Danicke S. (2006). Bioavailability of the Fusarium toxin deoxynivalenol (DON) from naturally contaminated wheat for the pig. Toxicol. Lett..

[7] Pinton P., Nougayrède J.-P., del Rio J.-C., Moreno C., Marin D.E., Ferrier L., Bracarense A.-P., Kolf-Clauw M., Oswald I.P. (2009). The food contaminant deoxynivalenol, decreases intestinal barrier permeability and reduces claudin expression. Toxicol. Appl. Pharm..

[8] Maresca M. (2013). From the gut to the brain: Journey and pathophysiological effects of the food-associated trichothecene mycotoxin deoxynivalenol. Toxins.

[9] Oswald I., Marin D., Bouhet S., Pinton P., Taranu I., Accensi F. (2005). Immunotoxicological risk of mycotoxins for domestic animals. Food Addit. Contam..

[10] Goyarts T., Grove N., Danicke S. (2006). Effects of the Fusarium toxin deoxynivalenol from naturally contaminated wheat given subchronically or as one single dose on the *in vivo* protein synthesis of peripheral blood lymphocytes and plasma proteins in the pig. Food Chem. Toxicol..

[11] Antonissen G., Martel A., Pasmans F., Ducatelle R., Verbrugghe E., Vandenbroucke V., Li S., Haesebrouck F., van Immerseel F., Croubels S. (2014). The impact of Fusarium mycotoxins on human and animal host susceptibility to infectious diseases. Toxins.

[12] Alexander J., Baines J., Bolger M., Duxbury J.M., Larsen J.C., Meyland I., Rao M.V., Renwick A.G., Schlatter J., Shephard G.S. (2011). Evaluation of certain contaminants in food: Seventy-second report of the Joint FAO/WHO Expert Committee on Food Additives. WHO Technical Report Series No. 959, 2011.

[13] Pinton P., Braicu C., Nougayrede J.P., Laffitte J., Taranu I., Oswald I.P. (2010). Deoxynivalenol impairs porcine intestinal barrier function and decreases the protein expression of Claudin-4 through a mitogen-activated protein kinase-dependent mechanism. J. Nutr..

[14] Pestka J.J. (2008). Mechanisms of deoxynivalenol-induced gene expression and apoptosis. Food. Addit. Contam. Part A Chem. Anal. Control Expo. Risk Assess..

[15] Bony S., Carcelen M., Olivier L., Devaux A. (2006). Genotoxicity assessment of deoxynivalenol in the Caco-2 cell line model using the Comet assay. Toxicol. Lett..

[16] Bianco G., Fontanella B., Severino L., Quaroni A., Autore G., Marzoco S. (2012). Nivalenol and Deoxynivalenol Affect Rat Intestinal Epithelial Cells: A Concentration Related Study. PLoS One.

[17] Wu C.C., Chen H.C., Chen S.J., Liu H.P., Hsieh Y.Y., Yu C.J., Tang R., Hsieh L.L., Yu J.S., Chang Y.S. (2008). Identification of collapsin response mediator protein-2 as a potential marker of colorectal carcinoma by comparative analysis of cancer cell secretomes. Proteomics.

[18] Goossens J., Pasmans F., Verbrugghe E., Vandenbroucke V., de Baere S., Meyer E., Haesebrouck F., de Backer P., Croubels S. (2012). Porcine intestinal epithelial barrier disruption by the Fusarium mycotoxins deoxynivalenol and T-2 toxin promotes transepithelial passage of doxycycline and paromomycin. BMC Vet. Res..

[19] Ke N., Wang X., Xu X., Abassi Y.A. (2011). The xCELLigence system for real-time and label-free monitoring of cell viability. Methods Mol. Biol..

[20] Diesing A.K., Nossol C., Dänicke S., Walk N., Post A., Kahlert S., Rothkötter H.J., Kluess J. (2011). Vulnerability of polarised intestinal porcine epithelial cells to mycotoxin deoxynivalenol depends on the route of application. PLoS One.

[21] Zhang C.L., Ren H.J., Li X.G., Sun D.L., Li N., Ming L. (2014). Modulation of Intestinal Epithelial Cell Proliferation, Migration, and Differentiation *in Vitro* by Astragalus Polysaccharides. PLoS One.

[22] Berridge M.V., Tan A.S. (1993). Characterization of the cellular reduction of 3-(4,5-dimethylthiazol-2-yl)-2,5-iphenyltetrazolium bromide (MTT): Subcellular localization, substrate dependence, and involvement of mitochondrial electron transport in MTT reduction. Arch. Biochem. Biophys..

[23] Garcia-Lorenzo A., Rodriguez-Pineiro A.M., Rodriguez-Berrocal F.J., de la Cadena M.P., Martinez-Zorzano V.S. (2012). Changes on the Caco-2 Secretome through Differentiation Analyzed by 2-D Differential In-Gel Electrophoresis (DIGE). Int. J. Mol. Sci..

[24] Marin D.E., Taranu I., Motiu M., Manda G. (2010). Effect of Lactobacillus feed supplement in deoxynivalenol intoxicated piglets. Arch. Zootech..

[25] Rosseli M., Finamore A., Garaguso I., Britti M.S., Mengheri E. (2003). Zinc oxide protects cultured enterocytes from the damage induced by *Escherichia coli*. J. Nutr..

